# The effects of different frequencies of rhythmic acoustic stimulation on gait stability in healthy elderly individuals: a pilot study

**DOI:** 10.1038/s41598-021-98953-2

**Published:** 2021-09-30

**Authors:** Roberta Minino, Emahnuel Troisi Lopez, Pierpaolo Sorrentino, Rosaria Rucco, Anna Lardone, Matteo Pesoli, Domenico Tafuri, Laura Mandolesi, Giuseppe Sorrentino, Marianna Liparoti

**Affiliations:** 1 Department of Motor Sciences and Wellness, University of Naples “Parthenope”, Naples, Italy; 2 Institut de Neuroscience des Systemès, Aix-Marseille University, Marseille, France; 3 Institute of Applied Sciences and Intelligent Systems, CNR, Pozzuoli, NA Italy; 4 Department of Social and Developmental Psychology, University of Rome “Sapienza”, Rome, Italy; 5 Department of Humanities Studies, University of Naples Federico II, Naples, Italy; 6 Institute for Diagnosis and Care, Hermitage Capodimonte, Naples, Italy

**Keywords:** Motor control, Neuroscience, Health care

## Abstract

The efficacy of rhythmic acoustic stimulation (RAS) to improve gait and balance in healthy elderly individuals is controversial. Our aim was to investigate, through 3D gait analysis, the effect of different types of RAS (fixed frequency and based on subject-specific cadence), using conventional gait parameters and the trunk displacement as readouts. Walking at a fixed frequency of 80 bpm, the subjects showed extended duration of gait cycle and increased gait variability while the same individuals, walking at a fixed frequency of 120 bpm, showed reduced trunk sway and gait cycle duration. With regard to the RAS at subject-specific frequencies, walking at 90% of the subject-specific average cadence did not significantly modify the gait parameters, except for the speed, which was reduced. In contrast, walking at 100% and 110% of the mean cadence caused increased stride length and a slight reduction of temporal parameters and trunk sway. In conclusion, this pilot study shows that using RAS at fixed frequencies might be an inappropriate strategy, as it is not adjusted to individual gait characteristics. On the other hand, RAS frequencies equal to or slightly higher than each subject's natural cadence seem to be beneficial for gait and stability.

## Introduction

Aging-related motor impairment stems from multiple factors, including bone loss, muscle atrophy and the decline of both the central and the peripheral nervous systems^1^. As a consequence, elderly people show reduced static and dynamic balance^2–4^, which results in higher risk of falls^5^, which has a major impact on public health^6–8^.

Different strategies have been adopted in order to increase the stability and reduce the risk of falls. Among these, sensory stimulation is a particularly promising approach^9–12^. In fact, the increase in sensory information, including auditory, visual or vibro-tactile stimulations, can improve static and dynamic balance, even for individuals with sensory and motor impairment^13^. Moreover, it has been widely demonstrated that an appropriate acoustic stimulation improves walking parameters in different pathological conditions, including Parkinson’s disease^14–16^, stroke^17,18^ and multiple sclerosis^19^. However, sensory stimulation, as a strategy to improve gait stability in the healthy elderly population, has been poorly investigated.

A large effort has been devoted to investigate how specific features of acoustic stimuli and rhythmic acoustic stimulation (RAS) affect walking. In fact, it is not known how to tune the RAS most effectively in order to maximise benefits in terms of balance and gait parameters, in either health or disease. Some studies adopted rhythmic stimulation based on fixed frequencies, (regardless of the natural cadence of each individual participant) ranging from 60 to 150 bpm^20,21^. For example, Dickstein et al. studied how walking synchronises to an acoustic stimulus at 60, 110, and 150 beats/min, showing that low frequencies display greater ease of synchronisation^21^. On the other hand, other authors investigated pacing frequencies based on each subject's average cadence, trying to increase or decrease the cadence of stimulation with respect to the subject-specific baseline (± 5%, ± 10%, ± 15%, ± 20% of the average cadence)^22–25^. For example, Yu and colleagues explored the effects of three different frequencies based on the cadence of each subject (i.e. 90%, 100% and 110% of the average cadence) on spatiotemporal gait parameters^22^. It was suggested that a faster stimulus affects multiple gait parameters, such as stride length, cadence and speed, as compared to the non-cued walking. Finally, in order to give a constraint without altering the pace speed, the possibility of using the subject's average cadence (100% of average cadence) was investigated^15,24,26^. However, the most effective stimulation frequency to improve balance and gait is yet to be defined. This is particularly relevant not only to inform rehabilitative strategies, but also to design devices based on RAS to be used in elderly people at high risk of falls.

It is noteworthy that many of the studies investigating the effects of RAS on gait have been focused selectively on the lower limbs, excluding the other body segments^27^. In order to maintain balance^28^, walking entails synergistic and coordinated motor adjustment of the upper limbs, shoulders, trunk and lower limbs^29^. Importantly, central balance control does not intervene on each muscle individually, but rather aims at the control of the centre of mass (COM) with segmental or sub-segmental adjustments left to hierarchically lower mechanisms^28,30,31^. Therefore, studying the static and dynamic balance of a population with higher risk of falling exclusively focusing on the lower limbs is highly limiting. Even considering the whole body, several measures have been developed to investigate the dynamic balance. Among the plethora of these indices, Maximum Lyapunov exponent, Harmonic Ratio, and Coefficients of Variability are commonly employed to quantify gait stability. However, several authors reported controversial results^32,33^ due to low predictive capability^34^ and several methodological discrepancies^34,35^.

Our group recently designed a synthetic—yet highly informative—measure of dynamic postural stability, the trunk displacement index (TDI), assessing the trunk displacement with respect to the centre of mass^31^. In particular, this measure, obtained from 3D-gait analysis (3D-GA) data, evaluates the ratio of the displacements of trunk and COM on three anatomical planes, conveying the overall motor performance. Considering that poor control of the COM increases the risk of falling, we hypothesise that the effectiveness of different acoustic stimuli on the gait stability could be evaluated by assessing the TDI value.

The aim of this exploratory study is to verify the effect of RAS in improving spatiotemporal gait parameters and stability in healthy elderly subjects. To this aim, we compared either fixed or variable acoustic stimulation frequencies on the kinematic parameters of the gait. In particular, we investigated the effects of fixed rhythmic stimulation frequencies (80 bpm and 120 bpm) on the spatiotemporal gait parameters and TDI, as measured from 3D-GA data. Furthermore, to test the hypothesis that the frequency of optimal gait stabilization is subject-specific, we evaluated the effects of lower (90%), equal (100%) or higher (110%) frequency than the average cadence of the subjects, in the same healthy elderly population. Finally, to further clarify the best frequency of RAS that benefits balance, we carried out a correlation analysis between gait parameters and TDI values.

## Methods

### Participants

Twenty-two elderly individuals (Table 1) were recruited from a day care centre for healthy elderly people, but only thirteen (6 males, 7 females) were included in the study, because nine of them did not meet the following inclusion criteria: *(i)* 65 to 85 years old; *(ii)* no skeletal, muscular and neurodegenerative disorders; *(iii)* no hearing impairment; *(iv)* Beck Depression Inventory (BDI)^36^ score < 13; *(v)* Mini Mental State Examination (MMSE) score > 23.80^37^; *(vi)* Frontal Assessment Battery (FAB) score > 12.03^38^. Written informed consent was obtained from all participants prior to participation. All methods were carried out in accordance with the Declaration of Helsinki guidelines. The study was approved by the Local Ethic Committee (University of Naples Federico II, n. 26/2020).

**Table 1:** Demographic, anthropometric and neuropsychological participants’ characteristics (mean ± standard deviation).

Participant characteristics	
**Demographic data**	
Ages (years)	73.4 (± 5.7)
Education (years)	11.6 (± 4.8)
Gender (m/f ratio)	6/7
**Anthropometric parameters**	
Weights	65.27 (± 7.57)
Heights	161.78 (± 8.89)
BMI	25.0 (± 2.8)
**Neuropsychological evaluation**	
MMSE (adjusted)	27.65 (± 1.85)
FAB	16.41 (± 1.66)
BDI	6.85 (± 3.98)

Weights in kilograms, heights in centimetres, body mass index (BMI), mini mental state examination (MMSE), frontal assessment battery (FAB), Beck’s depression inventory (BDI).

### Gait analysis assessment

#### Protocol

**Figure 1:**
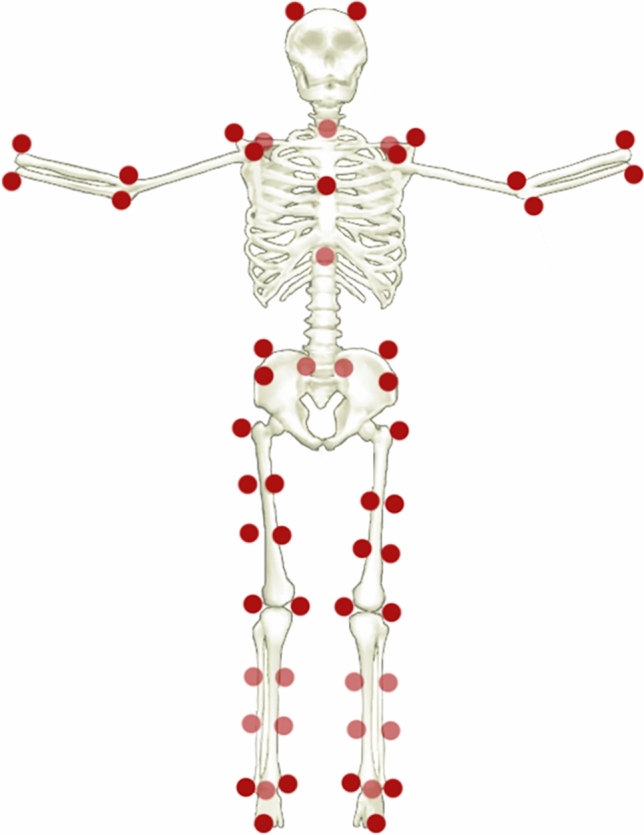
Marker set. Visual representation of the fifty-five markers positioned on the anatomical landmarks of feet, lower limb joints, pelvis, trunk, upper limb joints and head.

The 3D-GA was carried out in the Motion Analysis Laboratory of the University of Naples “Parthenope”. The 3D-GA data were acquired through a Stereophotogrammetric system, equipped with eight infrared cameras (ProReflex Unit-Qualisys Inc., Gothenburg, Sweden). In agreement with the modified Davis protocol^39^, 55 passive markers were positioned on each participant on anatomical landmarks of the feet, the lower limb joints, the pelvis and the trunk, as well as on the upper limb joints and on the head (Fig. 1). The data acquisition phase was carried out in two distinct times. During the first one, in order to calculate the natural cadence of each participant, the subjects were recorded at a self-selected speed, while they walked back and forth on a 10 m walkway. In the second phase, we recorded the participants’ walking in six experimental conditions: (1) simple walking (SW); (2) walking with RAS at fixed frequency, corresponding to 80 beats per minute (bpm); (3) walking with RAS at the fixed frequency of 120 bpm; (4) walking with RAS at a frequency corresponding to 90% of the participants’ cadence (90%-AC); (5) walking with RAS at a frequency corresponding to the 110% of the participants’ cadence (110%-AC); (6) walking with RAS at frequency corresponding (100%) to the subject-specific average cadence (AC); (Table 2). The RAS was emitted by a metronome (MA-1 Solo Metronome, Korg—UK). The acoustic stimulation was set to a tone of 440 Hz^40^ (such as a metronome tic) with an easily audible volume^41^. Prior to the acquisition phase, for the SW task, participants were instructed to walk on a 10-m walkway, back and forth, with their individual step speed, while prior to each RAS task, they had to walk on a 10-m walkway, back and forth, in time with the stimulation. For all conditions the subjects walked in an elliptical path, to allow subjects to follow the rhythm continuously. Each task was performed four consecutive times. After each task, the participants took a short break to both avoid fatigue and allow the experimenter to set the next stimulation frequency and provide the instruction of the new task. To reduce the learning and fatigue effect, the order of the acoustic stimuli was randomised. Although the participants were aware that they were being recorded during the task, they did not know when the recording started or finished. In fact, although subjects walked back and forth on the walkway during each task, the recordings were made only when they walked in the central part of the walkway to avoid possible confounding factors related to gait trajectory changes^42^. Moreover, before each experimental condition, we waited for the participant to synchronise to the provided stimulus for 10 s^20^. For each task four trials were recorded, each of them included two consecutive gait cycles, so for each task we collected eight gait cycles^43,44^. The data were processed using a tracking data software (Qualisys Track Manager by Qualisys AB, Göteborg, Sweden) and a software (Visual 3D by C-Motion Inc., Germantown, MD) to build a model of the skeleton^45,46^. In order to obtain a more reliable estimate, we calculated the average of eight gait cycles for each task.

**Table 2:** Frequencies based on average cadence of each participant measured in beats per minute (mean ± standard deviation).

Participants	100% AC frequency(bpm)	90% AC frequency (bpm)	110% AC frequency (bpm)
1	115	103	126
2	117	104	128
3	106	95	117
4	113	102	124
5	105	95	115
6	90	81	99
7	109	98	120
8	129	116	142
9	110	99	121
10	100	90	110
11	108	97	119
12	119	107	131
13	112	100	123
	110.23 (± 9.46)	99 (± 8.40)	121.15 (± 10.37)

The 100%AC is the frequency equal to the average cadence of each participant. The 90% AC is the frequency equal to 90% of the participants’ cadence. The 110% AC is the frequency equal to 110% of the participants’ cadence.

#### Spatiotemporal parameters

3D-GA has been used to obtain the temporal and spatial parameters of gait. The former included speed, stance time, swing time, cycle time and double limb support time (DLS). The latter included stride width and stride length. The variability coefficient (CV) (the ratio between standard deviation and average value × 100 (%) was calculated for all spatiotemporal parameters, except speed.

#### Trunk displacement index

In order to evaluate the dynamic stability, we calculated the TDI^31^, a newly described synthetic index. Several authors reported the importance of COM and trunk in maintaining stability during walking^32,35,47^. The making of the TDI stems from the evolutionistic perspective of the development of the upright posture and bipedal locomotion. Indeed, during this process the position of the trunk and the weight distribution changed^48,49^. This brought an increase in the complexity of the task of keeping balance, which sees an involvement of the cerebellum in controlling COM, exploiting vestibulospinal information of trunk verticality^28,30^. Since our aim was to build a synthetic measure, we only considered COM and trunk to build the TDI, deeming that the trunk should not be oscillating too much as compared to the COM.

To develop the TDI, we measured the trajectory of the centre of mass ($$COMt$$) during gait in the three-dimensional space and calculated its mean ($$\overline{COMt }$$) for each axis. Thereafter, we calculated the same three-dimensional trajectories for the upper trunk ($$Tt)$$. Subsequently, we calculated separately the distances between $$COMt$$ and $$Tt$$ from $$\overline{COMt }$$ , within in axis, for each registered frame of the gait cycle of the individual, obtaining two three-dimensional vectors of distances ($$COMd$$ and $$Td$$ respectively), as shown in Eqs. (1) and (2):

1 $$COMd=COMt-\overline{COMt }$$

2 $$Td=Tt-\overline{COMt }$$

Finally, to obtain a scalar conveying the three-dimensional displacements of the trunk, we performed two more steps. Firstly, we summed the norm of each vector of distances ($COMd$ and $Td$). Then, we calculated the ratio between those two values obtaining a dimensionless number, as shown in the following Eq. (3), representative of the relationship between the three-dimensional displacements of the trunk and the COM^31^.

3 $$TDI=\frac{\sum \Vert Td\Vert }{\sum \Vert COMd\Vert }$$

The obtained value is an estimation of how much the trunk oscillates with respect to the COM. Hence, higher values should be considered detrimental to stability.

### Statistical analysis

The statistical analysis was performed in Matlab (Mathworks, version R2018b). Each participant's parameter was normalised for body mass index (BMI) and the means and standard deviations of the gait parameters were calculated. Gait parameters were compared between SW and each cued condition, analysing two subsets separately. The first subset included the SW vs. the fixed frequencies (80 and 120 bpm); the second subset included the SW group vs. the variable frequencies (the 90%, 100%, 110%-AC groups).

To check the normal distribution of variables, the Shapiro–Wilk test was used. Given the heterogeneous distribution of the parameters and the small size of the sample, we performed nonparametric statistical testing. The Friedman test was used to investigate the differences within subgroups, while a two-sided Wilcoxon signed rank test was used to perform the pairwise comparison. Test statistic (W) and effect sizes (ESr) were reported for each comparison^50^. Spearman correlation tests were carried out to test the relationship between the TDI and the gait parameters individually. The p-values were corrected for multiple comparisons using the false discovery rate (FDR)^51^ and the statistical significance was set at p < 0.05.

## Results

We checked the group's ability to walk by following the administered stimuli, and the means and standard deviations of each experimental condition are shown in Table 3.

**Table 3:** Means and standard deviations of the group cadences for each experimental condition.

	100%-AC	90%-AC	110%-AC	80 bpm	120 bpm
Mean	109,221	98,931	119,613	84,950	118,207
DS	± 9520	± 8174	± 10,794	± 6325	± 3806

### RAS at fixed frequencies

The fixed frequencies and the SW parameters were compared through the Friedman test. The analysis of temporal parameters showed significant differences for speed (χ^2^(2) = 24.15, p < 0.001, pFDR = 0.004), stance time (χ^2^(2) = 24.15, p < 0.001, pFDR < 0.001), swing time (χ^2^(2) = 22.62, p < 0.001, pFDR < 0.001), cycle time (χ^2^(2) = 24.15, p < 0.001, pFDR < 0.001) and DLS time (χ^2^(2) = 20.46, p < 0.001, pFDR < 0.001). Among the spatial parameters, similarly to the low frequency results, the stride length (χ^2^(2) = 17.08, p < 0.001, pFDR < 0.001) was the only significant parameter. The variability analysis showed significant differences in the stance time CV (χ^2^(2) = 7.54, p = 0.023, pFDR = 0.036) and the swing time CV(χ^2^(2) = 8, p = 0.018, pFDR = 0.032). Finally, the stability analysis showed a significant difference in the TDI (χ^2^(2) = 12.92, p = 0.002, pFDR = 0.003).

#### Simple walking (SW) vs. 80 bpm RAS

**Figure 2:**
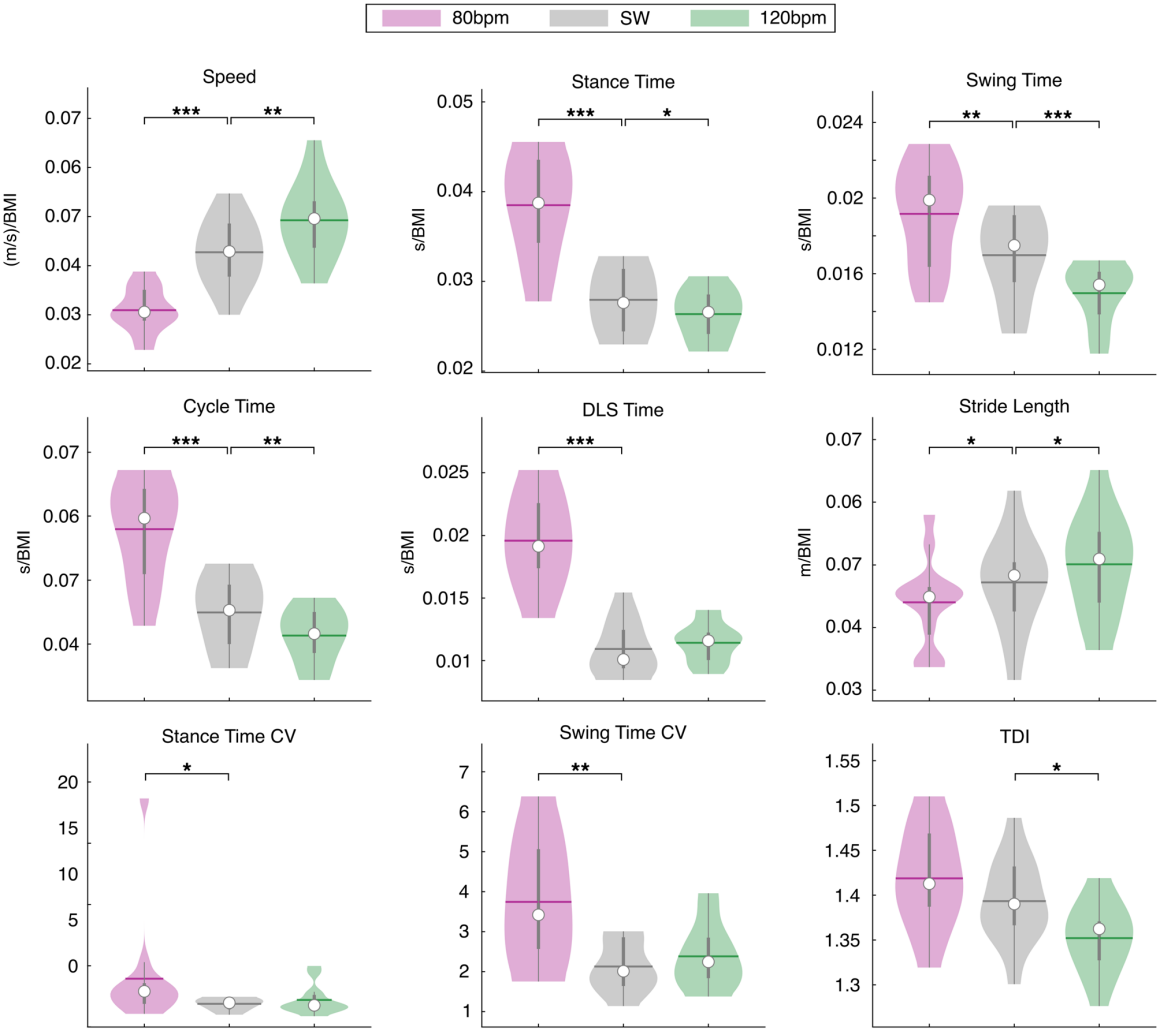
Spatiotemporal analysis of gait and Trunk Displacement Index (TDI). SW vs. RAS at variable frequencies. Violin plots of spatiotemporal parameters and TDI value. Comparison between simple walking (SW) and walking with RAS at variable frequencies based on the individual average cadence (AC) (SW; 90%-AC; 110%-AC; 100%-AC). DLS (double limb support). Significance p value: *p < 0.05, **p < 0.01, ***p < 0.001.

With regard to the temporal parameters, the walking at fixed frequency, i.e. 80 bpm, caused a statistically significant reduction of the speed (W = 91, p < 0.001, pFDR < 0.001, ESr = 1), and an increase of the stance time (W = − 91, p < 0.001, pFDR < 0.001, ESr = − 1), swing time (W = − 83, p = 0.002, pFDR = 0.004, ESr = − 0.91), cycle time (W = − 91, p < 0.001, pFDR < 0.001, ESr = − 1) and DLS time (W = − 91, p < 0.001, pFDR < 0.001, ESr = − 1), as compared to SW. For the spatial parameters, the 80 bpm fixed frequency caused a decrease of the stride length (W = 63, p = 0.027, pFDR = 0.034, ESr = 0.69). Furthermore, concerning the variability, this frequency of stimulation also caused increased stance time CV (W = − 67, p = 0.017, pFDR = 0.024, ESr = − 0.74) and swing time CV (W = − 77, p = 0.005, pFDR = 0.009, ESr = − 0.85). No significant difference was found in the TDI between these two conditions (Fig. 2).

#### Simple walking (SW) vs. 120 bpm RAS

The comparison between SW and walking with the RAS set at 120 bpm showed increased speed (W = − 89, p < 0.001, pFDR = 0.0012, ESr = − 0.98), and reduction of the stance time (W = 75, p = 0.006, pFDR = 0.0121, ESr = 82), swing time (W = 91, p < 0.001, pFDR < 0.001, ESr = 1) and cycle time (W = 89, p < 0.001, pFDR = 0.0012, ESr = 0.98). Moreover, our data showed increased stride length (W = − 71, p = 0.011, pFDR = 0.01625, ESr = − 0.78) and reduced TDI (W = 71, p = 0.011, pFDR = 0.01625, ESr = 0.78) (Fig. 2). Variability measures did not show any significant difference.

### RAS at variable frequencies (% of the basal cadence)

The comparison of temporal parameters among variable frequencies and SW showed significant differences in speed (χ^2^(3) = 28.58, p < 0.001, pFDR < 0.001), stance time (χ^2^(3) = 31.15, p < 0.001, pFDR < 0.001), swing time (χ^2^(3) = 25.15, p < 0.001, pFDR < 0.001), cycle time (χ^2^(3) = 33.46, p < 0.001, pFDR < 0.001) and DLS time (χ^2^(3) = 27.92, p < 0.001, pFDR < 0.001). Concerning the spatial parameters, the same analysis showed a difference only in the Stride Length (χ^2^(3) = 15, p = 0.002, pFDR = 0.004). No change was observed in variability. Finally, the analysis of the stability highlighted a difference in the TDI (χ^2^(3) = 15.65, p = 0.001, pFDR = 0.03).

#### Simple walking vs. 90%-AC

**Figure 3:**
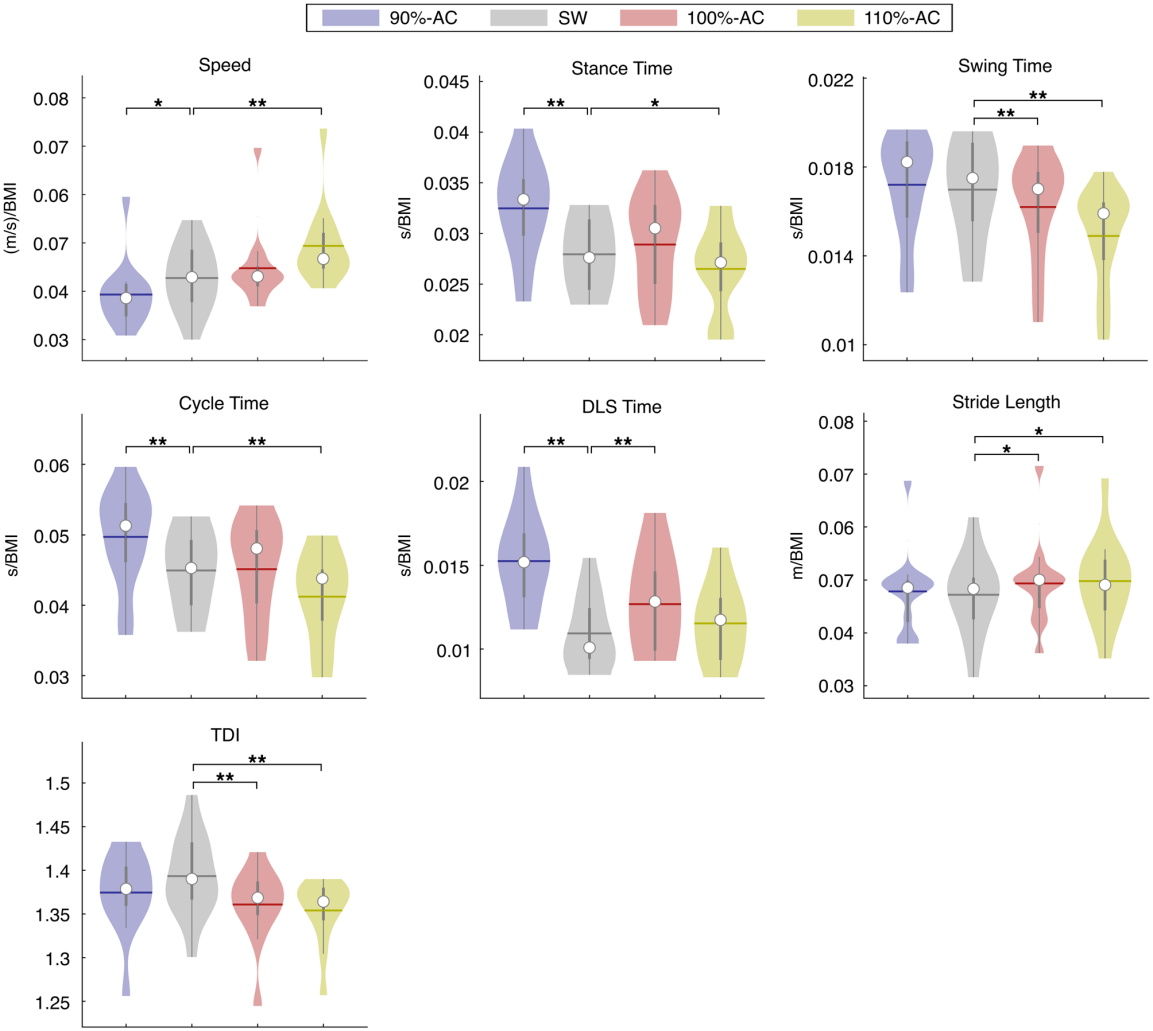
Spatiotemporal analysis of gait and Trunk Displacement Index (TDI). SW vs. RAS at fixed frequencies. Violin plots of spatiotemporal parameters and TDI value. Comparison between simple walking (SW) and walking with RAS at fixed frequencies expressed in beat per minute (bpm) (SW; 80 bpm; 120 bpm). DLS (double limb support), CV (coefficient of variability). Significance p value: *p < 0.05, **p < 0.01, ***p < 0.001.

The comparison between SW and 90%-AC showed differences in the temporal parameters. In particular, the reduced frequency caused a statistically significant decrease of the speed (W = 61, p = 0.033, pFDR = 0.049, ESr = 0.67) and an increase of the stance time (W = − 91, p < 0.001, pFDR = 0.001, ESr = − 1), cycle time (W = − 89, p < 0.001, pFDR = 0.002, ESr = − 0.98) and the DLS time (W = − 91, p < 0.001, pFDR = 0.001, ESr = − 1). Moreover, no significant differences were found in spatial parameters, coefficients of variability and in the TDI value in the comparison between these two conditions (Fig. 3).

#### Simple walking vs. 110%-AC

The results showed that 110%-AC affected all temporal parameters. In particular, it was documented a statistically significant increase of speed (W = − 85, p = 0.001, pFDR = 0.004, ESr = − 0.93) and decrease of the stance time (W = 63, p = 0.027, pFDR = 0.043, ESr = 0.69), the swing time (W = 91, p < 0.001, pFDR = 0.001, ESr = 1) and the cycle time (W = 91, p < 0.001, pFDR = 0.001, ESr = 1), compared to the SW. Concerning the spatial parameters, this frequency of stimulation caused a rise of the stride length (W = − 75, p = 0.006, pFDR = 0.012, ESr = − 0.82). Moreover, the results showed a reduction of the TDI compared to the SW (W = 81, p = 0.002, pFDR = 0.006, ESr = 0.89) (Fig. 3).

#### Simple walking vs. 100%-AC

The comparison between SW and 100%-AC showed, with regard to the temporal parameters, that the latter induced a significant decrease of the swing time (W = 77, p = 0.005, pFDR = 0.01, ESr = 0.85) and an increase of the DLS time (W = − 81, p = 0.002, pFDR = 0.006, ESr = − 0.89). About the spatial parameters, at 100%-AC it was observed an increased stride length (W = − 71, p = 0.011, pFDR = 0.018, ESr = − 0.78). Moreover, at the 100%-AC, the TDI was decreased as compared to SW (W = 81, p = 0.002, pFDR = 0.006, ESr = 0.89) (Fig. 3).

#### Correlation analysis

**Figure 4:**
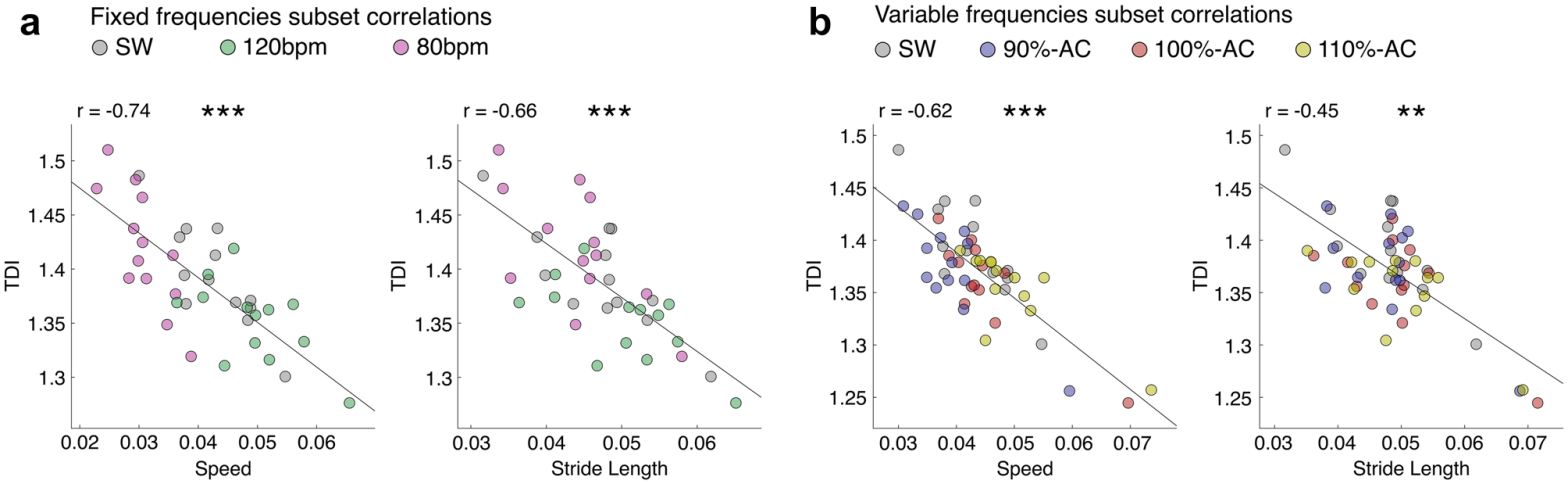
TDI and spatiotemporal gait parameters. Spearman correlation coefficient between trunk displacement index (TDI) and spatiotemporal gait parameters in simple walking (SW) vs. fixed and variable RAS frequencies separately. Average cadence (AC), beat per minute (bpm). Significance p value: *p < 0.05, **p < 0.01, ***p < 0.001.

To explore the relationship between the dynamic balance (expressed as TDI values) and the gait parameters, we performed a correlation analysis within the two groups, i.e. stimulated with both fixed (vs. SW) and variable (vs. SW) frequencies. In both experimental groups, a significant negative correlation between the TDI and both speed (fixed vs. SW: r = − 0.74, p < 0.001, pFDR < 0.001; variable vs. SW: r = − 0.62, p < 0.001, pFDR < 0.001) and stride length (fixed vs. SW: r = − 0.68, p < 0.001, pFDR < 0.001; variable vs. SW: r = − 0.45, p < 0.001, pFDR = 0.003) was found (Fig. 4).

## Discussion

In this pilot study, we investigated how different frequencies of the RAS may affect walking in healthy elderly subjects, assessing the spatiotemporal parameters (speed, stance time, stance time CV, swing time, swing time CV, cycle time, cycle time CV, double limb support time, double limb support time CV stride width, stride width CV, stride length and stride length CV) and the TDI, a measure of dynamic postural stability. Preliminary, in order to evaluate whether RAS at fixed frequencies were able to improve walking, we compared the basal walking with two fixed RAS frequencies (80 and 120 bpm).

Our results showed that the RAS at 80 bpm as compared to SW, induced a reduction in speed and an increase in stance time, swing time, cycle time and DLS time, indicating the ability of the subjects to synchronise to the stimulus. Furthermore, this frequency also caused a reduction in the stride length and increased stance time CV and swing time CV, suggesting greater instability. Our results are in agreement with Del Olmo and colleagues^20^ whereby in both PD and matched controls it was found that low cadence causes a reduction in stride length and increased parameters of gait variability. With regard to the comparison between the RAS at 120 bpm and SW, it was observed that the former increased the speed and the stride length with the consequent reduction of the stance, swing and cycle time. These results are in agreement with Ducharme et al.^52^, who examined several fixed frequencies (from 80 to 140 bpm). In particular, as in our case, they found an increase in speed, cadence and stride length during a stimulation at 120 bpm. On the one hand, this condition did not cause alterations in the DLS time. On the other hand, it induces a reduction of the TDI values (representing reduced oscillations of the trunk over the COM). Overall, these data suggest that the gait changes under the effect of the higher RAS should be regarded as beneficial for stability of the body as a whole. In conclusion, our results seem to suggest that RAS at low frequency (80 bpm) leads to greater instability as compared to that one at higher frequency (120 bpm), which in fact improves stability. However, these results need to be interpreted in relation to the specific gait features of our experimental sample. The range of fixed frequencies, used by many authors, varies from 60 to 150 bpm^20,21^. As this range is quite wide, the choice of fixed frequencies was based on data indicating that the average moderate cadence of a group of healthy adults is about 100 bpm^53–56^. Tudor-Locke et al^57^ categorised the cadence in a very large population into four categories: slow (60–79 steps/min), medium (80–99 steps/min), brisk (100–119 steps/min), and all forms of faster locomotion (≥ 120 steps/min). On this basis, to test the effects of RAS at fixed rates, we relied on a cadence of 100 bpm, as the frequency at the limits of moderate and brisk cadences, without deviating too much from the average cadence of the general population. However, in our sample this assumption turns out to be inaccurate, as our population had an average cadence of 110.23 steps/min, so the stimulation at 80 bpm was much lower than the basal cadence, unlike the 120 bpm stimulation, which was closer to the average cadence of the group. These results show that using RAS at fixed frequencies is not the most effective strategy because, not taking into account the individual cadence of the subject, sets the stimulations either too low or too high. The discrepancy of our sample compared to the general population could be due to the fact that we recruited it in a social centre where the subjects conducted an active life. In fact, it has been widely demonstrated that the average cadence is influenced by physical activity^58–60^.

In order to test the hypothesis that adjusting the RAS at subject-specific rates is more effective on gait stability, we evaluated on the same population also the effects of frequencies based on the subjects' natural cadence. We selected a frequency lower (90%), equal (100%) and higher (110%) than the average cadence of each subject^22–24^. The comparison of the temporal gait parameters recorded in SW and 90%-AC showed that the latter reduced the speed and increased the stance, cycle and DLS times. No change was evident for the spatial parameters, the coefficients of variability and the TDI. These results denoted a slight slowdown in the gait without impairment of the stability. The increase of the DLS time could be caused by a mechanism of synchronisation with the stimulus. We speculated that in order to adapt to the low frequency stimulus, the participants increased the time of the whole cycle by increasing the time spent in double stance, waiting for the next stimulus in a condition of higher stability (both feet on the ground instead of one as in the swing phase). The gait modifications induced by 90%-AC are in agreement with Willems et al. who analysed different frequencies of stimulations, both in parkinsonian patients and in healthy subjects, and found reduced speed and cadence and unmodified stride length^23^. Furthermore, the 110%-AC, as compared to SW, increased speed and stride length, while reduced the stance, swing and cycle times. Our findings are in accordance with Yu et al. which found increased speed, cadence and stride length in healthy young subjects, walking at 110%-AC, compared to baseline walking^22^. Moreover, no coefficient of variability was affected. Importantly, this frequency of stimulation lowered TDI values. It can be noted that the 110%-AC had the same effect as the fixed high cadence (120 bpm). This is caused by the matching of the frequencies in the two conditions. As previously explained, this is due to the fact that the choice of fixed frequencies was based on data from the literature which did not occur in our sample. Altogether, these results show that the gait changes occurring at 110%-AC are compatible with better control of the body sway and, consequently, improved body stability.

Finally, we compared the SW with the RAS at a frequency equal to the average cadence of each subject (100%-AC). The results highlighted that, by setting this frequency, the temporal parameters showed no significant changes, except for the swing and the DLS times, while for the spatial parameters, longer stride length was observed. Indeed, while the temporal parameters suggested entrainment with the RAS, the increase in stride length suggests better stability as confirmed by several studies^31,61,62^. The data also revealed higher DLS time which is commonly related to impaired stability and increased fall risk^63–65^. However, as in the case of the 90%-AC, we interpret the reduced swing time and the increased DLS time as a mechanism of synchronisation with the stimulus, and not as a loss of stability, which is in agreement with the observed reduction of the TDI. These results agree with the study of Arias et al.^24^ on the effects of rhythmic sensory stimulation on gait in parkinsonian patients and age-matched healthy controls. However, the authors also reported increased speed and reduced stride time variability. This partial discrepancy could be caused, among other factors, by the age difference of the cohorts.

The correlation study showed that the TDI correlated negatively with the speed. Indeed, the significant increase in speed, observed in both the 110%-AC and the 120 bpm stimulation, was associated with a lower TDI. These results suggest that higher speed—within a certain range—may enhance stability during the gait. On the contrary, when the walking speed decreased, we observed two different outcomes. In fact, when walking under the effect of the stimulation at 80 bpm, the individuals showed a slight TDI increase, while the TDI slightly decreased when the same subjects walked at 90%-AC. These results suggest that the presence of a RAS close to the natural individual cadence stabilises the posture during gait but when the stimulus imposes a further slowing down, the gait stability worsens. This is supported by observing the 100%-AC which showed no significant difference in speed, but a better stability—conveyed by the significant reduction of the TDI values. Unfortunately, we could not test if an increase of the TDI occurs even when the RAS induces a greater increase of gait speed.

Moreover, the TDI was negatively correlated to the stride length. In a similar line of thinking as before, a RAS close to the natural individual gait pace may increase stability during gait, which worsens when the individuals perform shorter steps. Our results agree with Osoba et al. who described that slow speed and short stride length is a cautious walking mechanism implemented to increase stability^66^. Furthermore, a clinical guide presented by Pirker and Katzenschlager on gait disorder in elderly people, states that ageing is related to a reduction in the step length and, consequently, in speed, highlighting the relationship between speed and the general health of the subjects^67^.

Some limitations of our study should be mentioned. First, the small sample size which made the study underpowered. This was due to the difficulty in finding and recruiting elderly subjects that were cognitively and physically healthy based on clinical and neuropsychological evaluation. Furthermore, although we aimed to continue the recruitment, the pandemic made it impossible. Another limitation is that the two high frequencies were quite similar to each other, hence we could not explore the effects of a stimulation with a frequency much higher than the average cadence of each subject, as we did for the low frequencies. Future research on the effects of RAS on the gait might identify the threshold values above which there is a worsening of the gait and a reduction in stability.

## Conclusions

The present pilot study suggests that the effects of RAS on gait depend on the frequency. In particular, our results show that using fixed frequencies may not be an appropriate strategy. In fact, not taking into account the individual gait characteristics may entail the application of stimuli that could be detrimental to stability. Although this hypothesis is supported by the data obtained in the case of the low fixed frequency (80 bpm), further investigation with a stimulation frequency higher than 120 bpm is warranted.

With regard to the variable frequencies, reducing the cadence to 90% does not seem to affect stability in any way, despite the slower gait cycle. However, a moderate increase of the cadence seems to be beneficial to gait and dynamic stability. Furthermore, we observed that similar effects were present when tuning the frequency of the stimulation to the individuals’ cadence. Finally, our preliminary study also seems to suggest that a moderate increase in speed and stride length improves the stability.

Further studies are needed to confirm our results and to identify the best setup of the RAS. This information may support classical rehabilitation with an effective sensory training, and help to develop preventive techniques to support walking and stability in people with a high risk of falling.
